# Copper and zinc concentrations in the uterine fluid and blood serum during the bovine estrous cycle

**Published:** 2012

**Authors:** Sayed Mortaza Alavi-Shoushtari, Siamak Asri Rezaie, Mozhgan Pak, Sajad Alizadeh, Roya Abedizadeh, Amir Khaki

**Affiliations:** *Department of Clinical sciences, Faculty of Veterinary Medicine, Urmia University, Urmia, Iran.*

**Keywords:** Copper, Zinc, Uterine fluid, Bovine

## Abstract

To investigate uterine and serum copper (Cu) and zinc (Zn) concentrations variation during the bovine estrus cycle , 232 blood and genital tract samples were collected from the abattoir in Urmia. The phase of the estrous cycle was determined by the examination of the ovaries and the uterine tonicity. Of the 46 samples selected for use in the study, 13 were pro-estrus, 10 estrus, 8 metestrus, and 15 diestrus. The uterus was incised and uterine fluid was collected by gentle scraping of the uterine mucosa with a curette. The total mean (± SEM) Cu concentrations in serum and uterine fluid samples, determined by spectrophotometry, were 66.1± 6.5 and 171.3 ± 33.2µg dL^-1^ respectively, which were significantly different, while total mean serum and uterine fluid Zn concentrations were 91.9 ± 5.4 and 291.6 ± 23.4 µg dL^-1^, which also showed a significant difference. The mean serum Cu values in different phases of the estrous cycle were not significantly different, while uterine fluid Cu content in pro-estrus and diestrus were significantly higher than those in estrus and metestrus, and were also significantly higher than those of the serum samples. The mean Zn value of serum samples at different stages of the cycle was not significantly different. The mean Zn value of the uterine fluid samples was also not significantly different in different stages, but in pro-estrus, metestrus, and in diestrus they were highly significantly different from those of the serum. These results showed that Cu concentrations in the uterine fluid vary at different stages of the cycle and are higher than those in the blood serum, but, the uterine Zn content does not vary during the estrous cycle and is much higher than those in the serum, that seems to be due to the secretory action of the uterine mucosa.

## Introduction

The uterus by creating a suitable environment for gamete survival and transport and early embryonic development has an important role in the reproduction of domestic animals. To do this task, the uterus should secrete a variety of substances. Information about uterine secretions and their changes during the different phases of the estrous cycle allows taking measures for providing the best environment for early embryo’s development until the time of its attachment to the uterine wall and placental formation. 

Copper is necessary for many enzymes like the Cu–Zn–superoxide-dismutase (SOD), which is involved in cell protection against free radicals. Copper is also needed for the cytochrome C oxidase that is responsible for energy supply and for cellular and humoral immunity.^1^ Elevated copper concentrations reduce oxidative processes and glucose consumption that may cause immotility and reduced viability of the spermatozoa.^1^^,^^2^ Copper is involved in hypophyseal receptors function and controls the release of LH from pituitary gland.^3^ Copper also affects female reproductive performance and its deficiency may result in structural and biochemical abnormalities in the fetus.^3^ The symptoms of copper deficiency^4^ and the functions of copper in the dairy cows have been described.^5^^,^^6^

Zinc is closely related to the cell biochemistry, physiology and morphology. It is involved in enzyme functions and protein and carbohydrate metabolism, and its concentration in the uterine secretion functions as an intra– and extracellular cation regulatory mechanism.^7^ Zinc plays a role as an activator in several enzyme systems and involves in cell replication and differentiation, particularly in nucleic acid metabolism.^8^

Ions, including Cu and Zn, move through the epithelial cells of the uterus into the lumen of the reproductive tract causing a concentration gradient which in turn helps in an osmotic gradient providing the driving force to transport water by osmosis out of the epithelial cells into the uterine lumen. Leese points out that ion concentration and their movement are essential for the regulation of enzyme activity and of the pH of the uterine fluid.^9^ The ionic composition of uterine fluid is apparently derived from a combination of ions from the blood and ions secreted from uterine epithelium.^10^

Despite the importance of Cu and Zn concentrations in the uterine fluid, and their role in gamete, zygote and early embryo development, there is little published information available on the Cu and Zn contents of the uterine fluid during the estrous cycle in cattle. 

This work was carried out: (1) to investigate changes in copper (Cu) and zinc (Zn) concentrations in the uterine fluid during the estrous cycle of the cow and (2) to compare them with concentrations in the blood serum during the estrous cycle to find any possible relationship between them.

## Materials and Methods

Genital tract and blood sample of 232 slaughtered cows with unknown history of reproduction and plain of nutrition were collected from abattoir in Urmia (37˚ 33΄ N, 45˚ 4΄ E) between November 2010 and June 2011. Blood samples were collected by jugular vein puncture in plain test tubes before slaughter. Samples were quickly transferred to the lab in a cold box. In an initial examination, immature, pregnant and abnormal genital samples or samples with hemolyzed blood samples were discarded. Genital tracts were examined to determine the stage of their cycles by examining the structures on their ovaries and by their uterine tonicity, as described by Noakes.^11^ Following phases were determined in 132 tested apparently normal cyclic genital tracts: 20 pro-estrous, 13 estrous, 19 metestrus and 80 diestrus. Serum samples were obtained by centrifuging the clotted blood at 3000 rpm for 10 minutes, and stored in Eppendorph microtubes at -20 ˚C until examination. 

Uterine fluid samples were collected by gentle scraping of the mucosa by a curette after incisions made in both uterine horns and stored in Eppendorph microtubes at -20 ˚C until examination. Samples with any abnormal endometrial appearance or discharge were discarded. A total number of 46 serum and uterine fluid samples were used in the study. The uterine fluid and blood serum Cu and Zn contents of the samples were determined by spectrophotometry procedure (Camspec 330 UK spectrophotometer) using commercial kits (Copper, Zinc Assay Kit, Elitech, France) after thawing the samples at room temperature. The uterine fluid samples were diluted (1:10) before estimation.

The data was analyzed by using SPSS software (version 16 for windows, SPSS Inc., Chicago, IL, USA) computer program. Statistic mean and standard error of mean (SEM) were calculated for each group and compared with the others by One-way ANOVA and Tukey test, the student t test was used for the comparison of the total means, and the significance was attributed at *p* ≤ 0.05.

## Results

The total mean (± SEM) Cu concentrations in serum and uterine fluid were 66.1 ± 6.5 and 171.3 ± 33.2 µg dL^-1^ respectively, which were significantly different (*p* = 0.004), while, total mean serum and uterine fluid Zn concent-rations were 91.9 ± 5.4 and 291.6 ± 23.4 µg dL^-1^, which also showed a significant (*p* = 0.000) difference (Table 1). 

**Table 1:** Total concentration of Cu and Zn in serum and uterine fluid samples (Mean ± SEM).

**Parameters**	**No.**	**Serum**	**Uterine fluid**	***p*** ** value**
**Cu (µg dL** ^-1^ **)**	46	66.1 ± 6.5*	171.3 ± 33.2	0.004
**Zn(µg dL** ^-1^ **)**	46	91.9 ± 5.4*	291.6 ± 23.4	0.000

* denotes differences between rows at *p* < 0.01 level.

The mean Cu (± SEM) values of serum samples were 50.9 ± 10.0, 60.6 ± 25.8, 72.5 ± 10.0 and 81.7 ± 18.6 µg dL^-1^ in pro-estrus , estrus, metestrus and diestrus respectively, while, the mean Cu values obtained for uterine fluid samples in these phases were 187.2 ± 47.7, 66.6 ± 25.8, 51.8 ± 11.6 and 355.9 ± 88.9 µg dL^-1^, respectively. The mean serum Cu values in different phases of the estrous cycle were not significantly different, while uterine fluid Cu content in pro-estrus was significantly different with those of metestrus (*p* = 0.024), and in diestrus with those of estrus (*p* = 0.018) and metestrus (*p* = 0.02). Uterine fluid Cu values in pro-estrus (*p* = 0.026) and diestrus (*p* = 0.019) were also significantly different from those of the serum samples (Table 2).

**Table 2:** Concentrations of Cu in serum and uterine fluid during different phases of the estrous cycle. (Mean ± SEM).

**Phases of the cycle**	**No.**	**Serum (µg dL** ^-1^ **)**	**Uterine fluid (µg dL** ^-1^ **)**
**Pro-estrus **	13	50.9 ± 10.0*	187.2 ± 47.7^a^
**Estrus**	10	60.6 ± 9.5	66.6 ± 25.8^ac^
**Metestrus**	8	72.5 ± 10.0	51.8 ± 11.6bce
**Diestrus**	15	81.7 ± 18.6*	355.9 ± 88.9adf

* denotes difference between rows at *p* < 0.05 level.

**abcdef:** Different letters denote statistically significant differences within the phases *p* < 0.05.

The total mean (± SEM) serum Zn value was calculated as 91.9 ± 5.4 µg dL^-1^ which was highly significantly different (*p* = 0.002) with those of the total uterine fluid Zn content of 291.6 ± 23.4 µg dL^-1^ (Table 3). The mean Zn values of serum sample obtained in pro-estrus, estrus, metestrus and diestrus were 94.9 ± 10.2, 94.2 ± 13.4, 78.7 ± 9.7, and 95.0 ± 10.1 µg dL^-1^ respectively, which were not significantly different. While, the mean Zn values of the uterine fluid samples were 304.2 ± 38.6, 216.9 ± 61.3, 307.5 ± 68.2, and 322.1 ± 33.0 µg dL^-1^, in pro-estrus , estrus, metestrus and diestrus respectively. The uterine fluid Zn values in different phases of the estrous cycle showed no significant difference, but in pro-estrus (*p* = 0.000), metestrus, (*p* = 0.009) and in diestrus (*p* = 0.000) they were highly significantly different from those of the serum.

**Table 3:** Concentrations of Zn in serum and uterine fluid during different phases of the estrous cycle (Mean ± SEM).

**Phases of the cycle**	**No.**	**Serum (µg dL** ^-1^ **)**	**Uterine fluid (µg dL** ^-1^ **)**
**Pro-estrus **	13	94.9 ± 10.2**	304.2 ± 38.6
**Estrus**	10	94.2 ± 13.4	216.9 ± 61.3
**Metestrus**	8	78.7 ± 9.1**	307.5 ± 68.2
**Diestrus**	15	95.0 ± 10.1**	322.1 ± 33.0

** denotes difference between rows at *p* < 0.01 level. No significant difference was observed within the columns.

## Discussion

Little published information is available concerning the concentration of Cu and Zn in the uterine fluid or their variations during the phases of the bovine estrous cycle. This work was an attempt to get some information in this field. Collecting the uterine secretions in the cow bears some problems. Flushing the uterus through the cervical canal and flushing the exposed uterus through a laparotomy section has been reported as techniques of collecting uterine discharges. The former has the problem of concentrating the flushing, and side effects of a surgical operation are the sequela of the latter. In addition, these techniques could be carried out only in experimental animals. The procedure of collecting uterine samples in this study has none of these problems, but here, we did not have recorded history of the age, breed and plain of nutrition of the animals before slaughter, which have been reported^12^^, ^^13^ to influence the serum Cu and Zn contents. By discarding immature genital tracts and by counting the corpora albicans on the ovaries, the age of the animal, though not included as a factor in our calculations, was considered to be in a range of 3-6 years at the time of selecting samples. The cows sampled in the slaughter house were mostly of cross-bred local breeds.

Reportedly, the mean copper in blood plasma of cattle is 1.26 ± 0.31 µg mL^-1^ (≡126 ± 31 µg dL^-1^) which may vary according to the state of reproduction, plain of nutrition and age of the animal, and regarded values between 57 to 19 µg dL^-1^ as lower normal range.^12^^,^^13^ The same authors have reported that the zinc value in cattle serum was between 80 to120 µg dL^-1^. These are in agreement with the results obtained in this study, although we only emphasized on the state of the reproduction. Also, our results showed that the sampled animals were not Cu or Zn deficient before slaughter. Baxter reported the value of 9 - 26 µmol L^-1^(≡ 57-164 µg dL^-1^) for the serum Cu content of the normal cattle, and regarded the figure of 9 µmol L^-1^(≡ 57 µg dL^-1^) as the minimum normal level. He has reported the plasma zinc content of normal cattle as 9-18 µmol L^-1^(≡ 41-82 µg dL^-1^), and considers the values less than 9 µmol L^-1^(41 µg dL^-1^) as abnormal.^14^ Our results fit well in the range of these values.

 Akhtar *et al.* comparing plasma Cu and Zn concentrations of anestrous with cyclic buffalo cows found that these parameters in anestrous cows are significantly lower than those in cyclic ones.^15^ They concluded that Cu and Zn deficiencies, by themselves or in combination, might be responsible for the anestrous state in the animals, and Cu and Zn supplementation could improve their situation. Copper and zinc supplementation of beef cows improves their pregnancy rates after artificial insemination (AI), and plasma Cu and Zn concentrations in supplemented cows are higher than those in controls.^16^ Furthermore, this supplementation reduces the incidence of uterine infections, embryonic loss and endometrial scar formation, leading to a better postpartum uterine involution and fertility.^14^ But, Griffiths *et al.* found no difference in mean open days and days to services in Cu and Zn supplemented dairy cows and the controls.^17^

Verdugo *et al.* studied *in vitro* effects of Cu and Zn on rat uterine musculature and found that these two cations had contradictory effect; Zn^++^ had suppressive effect while Cu^++^ was stimulatory.^18^ Ho *et al.* found that homozygote rats with genetic defects of Cu-Zn superoxide dismutase had a lower fertility rates than heterozygous normal rats, which was considered as a results of more embryonic loss.^19^ El-Hendy *et al.* observed that in rats fed with Zn deficient diets the weight gain is less than those in controls and have lower hematological parameters and serum Cu and Zn concentrations.^20^

Impairment of the reproductive performance in the female and spermaotgenesis in the male may be the symptoms of animal zinc deficiency,^21^ and zinc by exerting an antioxidant activity and kreatinizing the teat canal mucosa has an effect in preventing mastitis in the first week of lactation in dairy cows.^22^ Furthermore, zinc has an effect on postpartum fertility of the dairy cow, and exerts its action by reducing the effects of stress induced by udder infections and foot problems and by stimulating immunity system function.^22^

In this study mean uterine fluid Cu concentrations in diestrus and pro-estrus were higher than those in the serum while in estrus it was not different. This may be the effect of a long period of the uterus being under the dominance of progesterone during diestrum and its effect continue until pro-estrus , after the regression of the corpus luteum (CL), and before the elevation of plasma estradiol concentration occurs. This observed elevation at diestrus and the observation of no difference between serum and uterine fluid Cu in estrus suggest that progesterone may possibly have a role in increasing the serum and the uterine Cu concentrations, while, estrogens, as it could be expected, may have an opposite role.

Michaluk and Kochman reported that combination of Cu and GnRH in releasing FSH and LH from the anterior pituitary is more effective than that of the natural GnRH.^3^ This may be an explanation for the lower fertility in Cu deficient animals, but, is contrary to our results, because in estrus, in which the peak of FSH and LH occur, serum Cu content was at a lower level. It is possible that the surge of these gonadotropins have a decreasing effect on the regulators of Cu concentrations in serum and uterus.

In this study uterine fluid Zn concentrations in the phases of the cycle were more than that in the serum and in metestrus, diestrus and pro-estrus the difference was highly significant. These are the periods in which the uterus is under the dominance or effects of the dominance of progesterone, which suggests that progesterone by affecting the secretory function of the uterine mucosa elevates the zinc concentration in the uterine fluid. This needs to be confirmed by further investigations carried out on a bigger population of animals and in controlled situations with a detailed examination of hormonal changes. 

It can be concluded that the cyclical changes in serum and uterine tissues leads to elevation of uterine luminal fluid copper and zinc concentrations which are at high levels at pro- estrus and diestrus phases of the estrous cycle. These ions, in parallel with variations in other substances present in the uterine fluid during the estrous cycle, possibly take part in providing a proper environment for the embryo to grow. 
